# GeneTrailExpress: a web-based pipeline for the statistical evaluation of microarray experiments

**DOI:** 10.1186/1471-2105-9-552

**Published:** 2008-12-22

**Authors:** Andreas Keller, Christina Backes, Maher Al-Awadhi, Andreas Gerasch, Jan Küntzer, Oliver Kohlbacher, Michael Kaufmann, Hans-Peter Lenhof

**Affiliations:** 1 Center for Bioinformatics, Saarland University, Saarbrücken, Germany; 2 Wilhelm Schickard Institute for Computer Science, Eberhard Karls University, Tübingen, Germany

## Abstract

**Background:**

High-throughput methods that allow for measuring the expression of thousands of genes or proteins simultaneously have opened new avenues for studying biochemical processes. While the noisiness of the data necessitates an extensive pre-processing of the raw data, the high dimensionality requires effective statistical analysis methods that facilitate the identification of crucial biological features and relations. For these reasons, the evaluation and interpretation of expression data is a complex, labor-intensive multi-step process. While a variety of tools for normalizing, analysing, or visualizing expression profiles has been developed in the last years, most of these tools offer only functionality for accomplishing certain steps of the evaluation pipeline.

**Results:**

Here, we present a web-based toolbox that provides rich functionality for all steps of the evaluation pipeline. Our tool GeneTrailExpress offers besides standard normalization procedures powerful statistical analysis methods for studying a large variety of biological categories and pathways. Furthermore, an integrated graph visualization tool, BiNA, enables the user to draw the relevant biological pathways applying cutting-edge graph-layout algorithms.

**Conclusion:**

Our gene expression toolbox with its interactive visualization of the pathways and the expression values projected onto the nodes will simplify the analysis and interpretation of biochemical pathways considerably.

## Background

Recent biotechnological advances provide the basis for high-throughput techniques that allow for measuring the expression of thousands of genes or proteins simultaneously. Both, the sheer size of the resulting data sets and its noisiness necessitate powerful automatic procedures for normalizing and evaluating these expression profiles. cDNA microarrays that allow for quantifying the expression levels of a wide variety of transcripts have become one of the most important experimental data source in the life sciences. Usually, transcript levels are measured under different conditions, resulting in two or more sets of expression profiles that have to be compared and analyzed in order to detect differentially expressed genes. Thereby, biochemical categories and pathways that exhibit different expression activities and thus different biochemical behavior can be detected.

For the statistical evaluation of gene sets, many stand-alone as well as web-based tools have been implemented over the past years [1]. The long list of published programs includes FatiGO [2], BiNGO [3], and GOstat [4] that analyze only enriched Gene Ontologies [5]. For microarry data, ErmineJ [6], CRSD [7], or GSEA-P [8] have been proposed. Other tools allow for the analysis of arbitrary experimental data (e.g. WebGestalt [9], Babelomics [10], or GeneTrail). Another class of approaches focuses on the pre-processing of microarray data and provides only basic statistical analysis, but does no offer methods for gene set enrichment analysis: PMmA [11] was one of the first tools for the detection of differentially expressed genes. The program NMPP [12] is tailored for the pre-processing of self-designed NimbleGen microarray data. Other tools, as AMDA [13] offer clustering methods and functional annotation of the differentially regulated genes. More examples of tools focusing on preprocessing and basic statistical evaluation are ArrayPipe [14], one of the first web-based application, or GEPAS [15], which provides clustering methods and can correlate its results to diverse clinical outcomes. Most recently, Morris et al. [16] described a comprehensive collection of perl modules for microarray management and analysis. However, none of these tools provide a dynamic graphical representation of the detected pathways. This has to be done manually using one of the existing network visualization tools. One of the most popular visualizers with a large user and developer base is Cytoscape [17], which also offers a plug-in architecture allowing to extend the functionality, e.g., for integrating data analysis methods. Other visualization tools for biological interaction data are VisANT [18], which has been designed specifically for the integrative visual data-mining of biological pathways, and OSPREY [19], which has been developed to explore large networks.

Here, we present the first framework that integrates data retrieval, pre-processing, gene set enrichment analysis, and network visualization. Our tool, called GeneTrailExpress (GTXP), represents a pipeline tailored for mining information from microarray experiments that offers rich functionality for all crucial steps of microarray evaluation. Notably, the gene set analysis of GTXP relies on our tool GeneTrail [20].

## Results and Discussion

**Figure 1:**
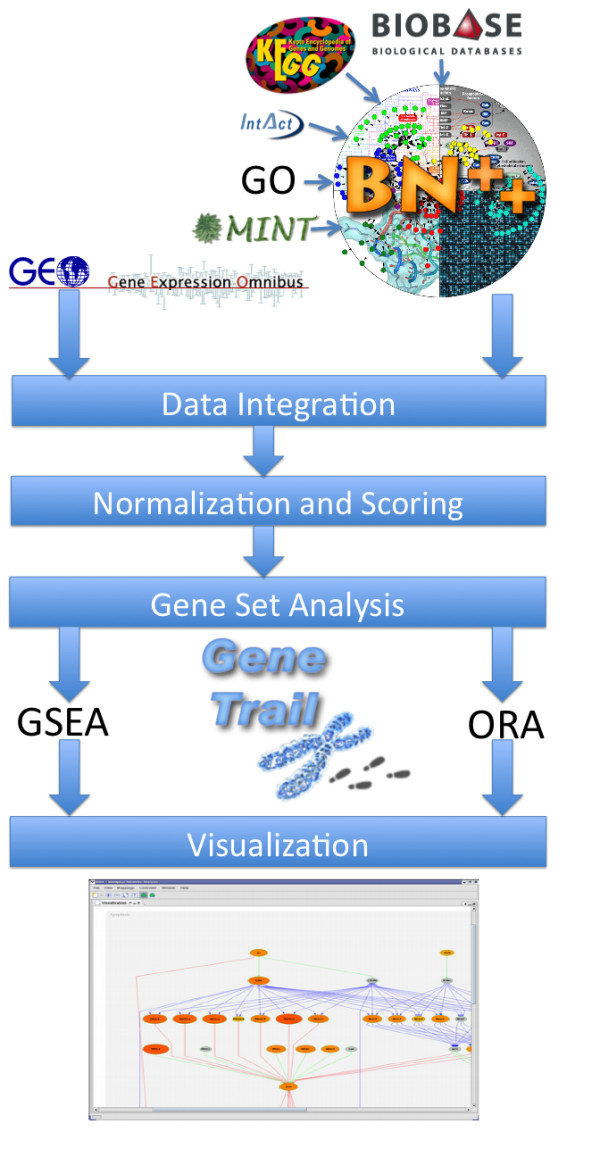
**GTXPs Workflow**.

Our web-based application GeneTrailExpress integrates all steps of a microarray analysis pipeline, as the workflow shown in Figure 1 outlines. GTXP guides the user through data retrieval, normalization, gene scoring, and the selection of biological categories for gene set analysis. After the gene set analysis has been carried out, the results are presented as a list of significant categories and pathways. Finally, the computed pathways can be visualized using a novel graph visualization tool called BiNA (Biological Networks Analyzer).

### Data integration

To perform gene set analyses, a variety of biochemical data extracted from heterogeneous databases is required, including regulatory and metabolic pathways from KEGG [21] and TRANSPATH [22], Gene Ontologies (GO) [5], and many more. Since GTXP imports most of these data sets from the biochemical network library BN++ [23,24] and the underlying database BNDB [25], the user only needs to load up the expression profiles to be analyzed. To this end, our tool offers a database connection to the NCBI Gene Expression Omnibus (GEO) [26]. Of course, the user can also upload his own flatfiles containing expression profiles.

### Pre-processing

For the different types of analyses, including normalization and gene scoring, various statistical methods are offered. To this end, we implemented a comprehensive C++ module that handles the statistical pre-processing of the expression profiles. Several normalization techniques are provided, as mean value normalization, median value normalization, or a normalization of mean and variance. The distributions of expression values before and after normalization are presented via bar charts.

Furthermore, several scoring functions for the computation of the differential expression are available: mean fold-change, median fold-change, unpaired t-test, paired t-test, Wilcoxon Mann-Whitney test, ANOVA, and Wilcoxon Rank-Sum test. The distribution of resulting scores is shown as a histogram.

Additionally, a list of all transcripts sorted by their score is generated. A brief summary on the scoring methods and application prerequisites can be found on the GTXP web interface. To test the stability and correctness of the implemented statistical tests, we cross-checked the results of GTXP with those of *R*, a widely used programming language for statistical computations.

### Gene Set Analysis

For the statistical evaluation of gene sets we apply our gene set analysis tool GeneTaril [20] that offers both common statistical approaches. The first method, the so-called "Over-Representation Analysis" (ORA), compares the set of interest to a reference set. When considering a certain biochemical category as a GO term, ORA tries to detect if this category is over-represented or under-represented in the respective gene set and computes its significance either by Hypergeometric test or by Fisher's test. The second method, which is cutoff-free, is called "Gene Set Enrichment Analysis" (GSEA). Here, the input set is sorted by some specific criteria (e.g., gene expression values). When considering an arbitrary functional category, GSEA tests if the genes in the set that belong to the category are randomly distributed or accumulated on top or on bottom of the sorted input list. While other tools estimate the GSEA p-values by non-parametric permutation tests, GeneTrail computes exact p-values by an efficient dynamic programming algorithm [27]. For a more precise description of both methods, GSEA and ORA, we refer to [20]. Other strengths of GeneTrail include the support of many organisms (among others *Homo sapiens*, *Mus musculus*, *Arabidopsis thaliana *and *Staphylococcus aureus*) and many biological categories (among others KEGG and TRANSPATH pathways, Gene Ontologies, transcription factors from TRANSFAC and sequence analyses). To integrate the diversity of data is realized by using the biochemical network library BN++ [23,24]. As comprehensive data source, BN++ can grasp a plenty of information of the underlying database BNDB [25].

GTXP enables the user to carry out GSEA and ORA analyses by including GeneTrail. For GSEA, the entire sorted gene list is used as input. For ORA, the gene list has to be separated in a test and a reference set. To this end, our tool provides different options: the user can decide to take the first *x *genes in the list, the first *x *percent of genes, or all genes with a score above or below a threshold as test set. In each case, the reference set contains all genes that are not included in the test set. For both gene set analysis approaches, GSEA and ORA, the biological categories to be analyzed can be chosen via a menu. After the gene set analysis has been carried out, the significant catgories are listed, sorted by the respective p-values.

### Network Visualization using BiNA

As discussed in the Background section, several tools for network visualization have been published in the last decade. We have developed BiNA, the Biological Network Analyzer, a visual analytics tool for biochemical networks. While a detailed description of BiNA is beyond the scope of this work, we will sketch its architecture and highlight its special features that are the reason for using BiNA in this project. BiNA consists of two parts, the platform and a plugin system. While the platform as central element of BiNA contains the graphical user interface and many common utilities, it does not have any possibilities for displaying or analyzing networks. For this task, we developed a powerful plugin structure, which plays an important role, both for the visualization of networks and also for the integration of BiNA into the BN++ framework. Besides the standard Java version, we also implemented a Java Webstart of BiNA allowing the seamless integration into websites.

BiNA builds upon our integrative system BN++ and the underlying comprehensive data warehouse BNDB. This warehouse system ensures a full semantic integration of many databases, including KEGG and TRANSPATH. Since GeneTrail relies on the same data warehouse system, the usage of BiNA ensures that the user gets visual representations of exactly the data that are analyzed by our gene set analysis tool. Since GTXP uses the Webstart version of BiNA, GeneTrail adds for each significant network a link on the results page. By following this link, the user directly generates a visualization of the respective network. To integrate the pathway data, we equipped BiNA with an SQLite interface to the BN++ database BNDB. If a pathway visualization is started for the first time, BiNA and all available topological network information are downloaded (about 40 MB) and stored on the local hard drive. Whenever BiNA is used again, a version control is carried out ensuring that the newest version of BiNA and the pathway topology information are available on the local disk. Thereby, an efficient visualization is guaranteed, even if the respective networks are large.

**Figure 2:**
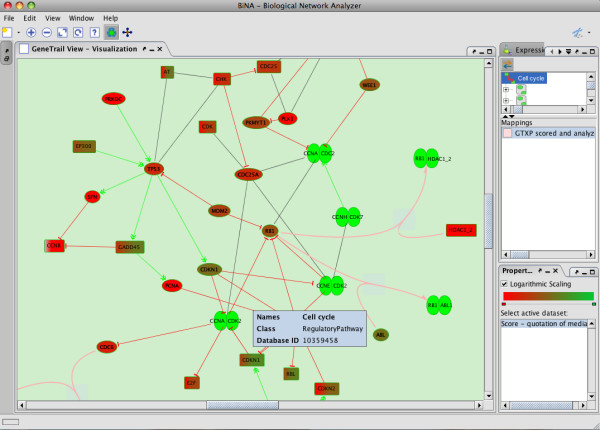
**BiNA GUI**. For the gene expression omnibus data set GDS1312, containing human lung cancer samples and normal controls, the result of the cell cycle pathway is sown. The performed gene set enrichment analysis computed a p-value of 0.0074, providing evidence for a clear up-regulation of the cell cycle in lung cancer. All genes are colored with respect to their over-expression, the tale green complexes correspond to protein complexes.

A key feature of BiNA is the comprehensive set of available graph layout algorithms. It includes most standard graph layouts (e.g., organic, circular, and hierarchical), but, in addition, also provides biologically inspired graph layouts, implementing the drawing conventions common in textbooks and allowing for a dynamical visualization of the networks using the static KEGG layout information. Moreover, BiNA provides convenient interactive analysis and navigation capabilities. Among others, BiNA allows to map arbitrary scalar data, like expression data, onto the biological networks. If a significant network is visualized by BiNA, the genes on the path are directly colored by their scores, facilitating the interpretation of the statistical evaluation. Figure 2 shows BiNA's graphical user interface visualizing a real biological example. A GSEA of lung cancer expression data reveals overexpression of lung cancer genes in the Cell Cycle, indicated by the red-colored genes.

## Conclusion

In this study, we present GeneTrailExpress, a toolbox that helps researchers to analyze and interpret expression data. The user is intuitively guided through all analysis steps of the pipeline. A main strength of our application is the integrated graph visualization tool that enables the user to draw the relevant biological pathways applying cutting-edge graph-layout algorithms. This interactive visualization of the pathways with the expression values projected onto the nodes facilitates the interpretation of significant findings considerably.

## Authors' contributions

AK and CB implemented the gene set enrichment method, AK contributed in writing the manuscript. MA implemented the web interface and the pre-processing. AG developed the BiNA tool. JK contributed to the data integration. MK, OK, and HPL are the senior authors and wrote the manuscript.

## Availability and requirements

Project name: GeneTrailExpress

Project homepage: 

Operating system: Platform independent

Programming language: Java, C++, php

Other requirements: JavaWS version 1.6 or higher
